# Building pre-service teachers’ resilience through Service-Learning: an explanatory sequential mixed methods study

**DOI:** 10.3389/fpsyg.2025.1568476

**Published:** 2025-04-15

**Authors:** Marta Sánchez-Jiménez, María Maravé-Vivas, Jesús Gil-Gómez, Celina Salvador-Garcia

**Affiliations:** ^1^CEIP Vil·la Romana, Valencia, Spain; ^2^Universitat Jaume I, Castellón de la Plana, Spain; ^3^Department of Education and Specific Didactics, Universitat Jaume I, Castellón de la Plana, Spain; ^4^Department of Pedagogy and Didactics of Social Sciences, Language and Literature, Universitat Jaume I, Castellón de la Plana, Spain

**Keywords:** Service-Learning, resilience, mixed methods, higher education, physical education, teacher education

## Abstract

University education should not be limited to mere instruction in professional skills but should foster the holistic development of students. This approach is particularly necessary in a changing society marked by rapid technological advances, labor transformations and global challenges. Therefore, higher education must offer students the opportunity to develop strategies to protect and improve their mental health. Within the concept of mental health is resilience, which enables the mobilization of internal and external resources to cope with and learn from challenging situations, managing change and uncertainty more effectively. Using a mixed methodological approach based on an explanatory sequential design, the present study aims to determine the effect of Service-Learning on the levels of resilience of students enrolled in the degree of Physical Activity and Sport Sciences at the Universitat Jaume I in Spain. To this end, the Resilience Questionnaire for Adults (RQA) was administered before and after a Service-Learning program. The reflection diaries written by the participants (*n* = 54) were analyzed too. The quantitative results show an improvement, which is significant for the “adaptative” and “tolerance” components of resilience, while the qualitative phase allows us to understand these results. In general, the findings point to the development of resilience among participants, which is a result consistent with previous literature suggesting that Service-Learning can be an adequate approach to foster the acquisition of skills present in the resilience construct, while favoring learning and fostering social compromise.

## Introduction

In line with Bauman’s (2003) perspective, we live in a changing, uncertain and increasingly unpredictable liquid society. He uses the metaphor of liquidity to highlight the transitory and volatile condition of today’s society. This society, marked by global challenges such as the aftermath of the pandemic, the rapid technological evolution and the transformation of the profile of the student body and the professions for which it is being prepared, puts great pressure on individuals, thus highlighting the importance of mental health. In this sense, Sohn (2022) states that promoting mental health is essential for people’s well-being and the proper functioning of our society. Faced with this reality, universities have a responsibility to address social inequality by training resilient, critical, sensitive and proactive individuals (Shephard and Egan, 2018). This university transformation represents an opportunity to integrate new methodological approaches that not only facilitate knowledge acquisition (Chiva-Bartoll and Gil-Gómez, 2018) but also the development of capacities associated with mental health, such as resilience. In this context, Masten (2001) states that fostering resilience in students allows them to face challenges with greater confidence and adaptability, preparing them to overcome academic difficulties and handle adversity in life. Prioritizing mental health in the educational environment is paramount, both for the well-being of students and for their academic success and their ability to cope with a changing and complex world (Fazel et al., 2014).

Within this framework, Service-Learning (SL) emerges as a pedagogical model that may contribute to the development of a range of competencies among university students (Salam et al., 2019). In addition to this, SL strengthens students’ capacity to adapt to and face challenges (Fletcher and Sarkar, 2013), which are essential skills present in the resilience construct (Prince-Embury and Saklofske, 2014). This is because SL is an experiential approach that combines student academic learning with community service (Bringle and Clayton, 2021), all accompanied by reflection processes. Particularly, students engage in a hands-on experience in order to address a community need. Such an experience is based on five pillars (i.e., investigation of a problem or issue; preparation and planning; action; reciprocity; and reflection), which should underpin every SL program (Chambers and Lavery, 2022).

Connecting theory and practice is essential to promote student learning, given that the application of curricular knowledge in real contexts can lead to the acquisition of skills (Gil-Gómez and Martí-Contreras, 2024); and SL emerges as an adequate opportunity to bridge the theory-practice gap (Resch and Schrittesser, 2023). Likewise, according to literature, intervening and adapting actions to the demands of a specific group facilitates the promotion of reflective, critical and resilient skills among students (Deeley, 2016; Shephard and Egan, 2018), while also overcoming prejudices (Domangue and Carson, 2008). By integrating the experience of community service with the learning process, SL gives participants the opportunity to develop crucial skills to face challenges (Bringle et al., 2006; Shephard and Egan, 2018); fostering the ability to mobilize the knowledge and skills necessary to respond and adapt to various challenges in their natural, social and personal environment (Simsek, 2020).

Due to the multiple benefits it may come with, SL is well established in the of higher education arena, including the field of Physical Activity and Sport (Chiva-Bartoll and Fernández-Rio, 2022; Pérez-Ordás et al., 2021). Experiences carried out in this context may focus, among others, on the promotion of healthy and active lifestyles, as well as on the creation of an inclusive context of interaction, where various groups recognize each other and participate as equals (Fernández-Cabrera et al., 2021). In this way, SL is positioned as a relevant tool given that it may trigger a comprehensive development of university students (Mayor Paredes, 2018). It entails offering valuable experiences that transcend the classroom, contributing to the training of more complete professionals who are aware of their potential impact on society (Chiva-Bartoll et al., 2020). Literature suggests that SL programs allow students to acquire management and problem-solving skills, academic performance, and critical thinking (Yorio and Ye, 2012), as well as the development of personal skills (i.e., self-esteem, self-recognition, motivation, and self-efficacy), social competences (i.e., interpersonal skills, social relations and the ability to reach agreements) and cultural and civic competences (i.e., understanding diversity, transformation of beliefs and social responsibility) (Cañadas, 2021). Thus, SL programs offer the opportunity to accept and become sensitive to diversity, to modify preconceived ideas and to adapt to the needs of the community, increasing self-efficacy and adaptability, which are all aspects included in the resilience construct (Prince-Embury and Saklofske, 2014).

As has been explained, the scientific literature reports how SL programs positively influence indicators related to the resilience construct (adaptability, sensitivity, tolerance and self-efficacy, among others). This article expands on this line, entering a novel field such as the influence that this approach may have on resilience. Resilience is understood as the dynamic and multidimensional capacity of people to adapt and recover effectively from adversity (Masten, 2001). This concept implies not only resistance to difficult situations, but also the process of learning and growing from these challenging experiences (Fletcher and Sarkar, 2013). Resilience does not consist of the absence of difficulties, but rather the intrinsic capacity of people to mobilize resources that allow them to face and overcome adversity (Bonanno et al., 2004). Masten (2001) highlights the dynamic nature of resilience, which is influenced by personal, social and contextual factors. According to this author, personal factors are linked to individual characteristics that help a person cope with and overcome adversity, such as optimism, self-efficacy, and emotional regulation. Social factors, such as family support, friendship networks, and intimate relationships, focus on providing emotional and practical resources in times of need. Contextual factors refer to the conditions and circumstances of the environment that can facilitate or hinder the development of resilience, such as the community environment, access to resources, and public policies. These intrinsic factors do not operate in isolation but interact with each other to strengthen an individual’s resilience. In addition, they can be developed and strengthened over time through experiences and learning (Alonso-Tapia et al., 2017).

In this sense, resilience is presented as a crucial phenomenon for psychological well-being and mental health, as it provides individuals with the necessary tools to face life’s challenges and maintain emotional balance (Bonanno et al., 2004; Fletcher and Sarkar, 2013). Promoting resilience is essential to strengthen people’s ability to face difficulties and develop a sense of self-efficacy (Masten and Motti-Stefanidi, 2020). Along these lines, it has been found that people participating in SL programs collaborating with groups of people with functional diversity show a higher level of self-efficacy (Reina et al., 2016) and stimulation of their resilience capacity (Fernández-Cabrera et al., 2021). At this point, it is important to delve deeper into resilience. Prince-Embury (2006) and Prince-Embury and Saklofske (2014) argue that the construct is composed of three components (Table 1).

**Table 1:** Components and elements that make up the resilience construct.

Components and definition	Associated indicators	Definition
Mastery: a sense of competence or efficacy driven by an innate curiosity, which contributes to the development of problem-solving skills.	Optimism (Sabouripour and Roslan, 2015)	Set of positive attitudes regarding the future, the world in general and one’s own life in particular (Prince-Embury, 2006).
	Self-efficacy (Keye and Pidgeon, 2013)	The way in which a person masters their environment and faces obstacles, associated with the development of problem-solving strategies.
	Adaptability (Bryan et al., 2015)	Ability to be receptive to criticism, to learn from mistakes and to ask others for help.
Relational sense: the way in which the individual relates to others in adverse situations (Prince-Embury, 2008).	Sense of trust	The degree to which an individual can be authentic with others and find them trustworthy.
	Perception of access to support	The individual’s belief that there are others they can turn to when faced with adversity.
	Comfort	The degree to which a person can be in the presence of others without feeling discomfort or anxiety.
	Tolerance	Regarded as the person’s belief that they can safely express their differences.
Emotional reactivity: the individual’s ability to modulate and regulate their emotional responses (Prince-Embury and Saklofske, 2014).	Recovery	Ability to recover from emotional arousal or disturbance of balance.
	Sensitivity	Emotional reactivity of individuals to environmental demands.
	Deterioration	The degree to which a person is able to maintain emotional balance when aroused.

Prepared by the authors based on Prince-Embury (2006) and Prince-Embury and Saklofske (2014).

Once the construct is presented, it is worth noting that there is a notable lack of intervention programs focused on examining resilience within the field of higher education and the ages corresponding to this stage (Gillham et al., 2007; Brewer et al., 2019; Masten, 2014). The intersection between SL and resilience opens an interesting field of exploration in the educational and social spheres in light of the effects pointed out in the literature on variables related to the construct.

## Materials and methods

### Objective, hypothesis and research question

The general objective of this article is to analyse the effects produced by a SL program in a subject of the degree of Physical Activity and Sports Sciences regarding the resilience of the students. The research was carried out using a mixed methods approach, so hypotheses and research questions were formulated. The hypothesis for the quantitative part of this study was: the application of SL in a university subject of the Physical Activity and Sports Sciences degree will produce significant improvements (*p* < 0.05) in the resilience levels of the participating students. In case the hypothesis is confirmed, in order to explain such changes in the dependent variable (resilience), the following research question was posed for the qualitative part of the study, as it would be instrumental to deepen and understand the results previously obtained through its answer: How does SL affect the dimensions of resilience?

### Design

**Figure 1:**
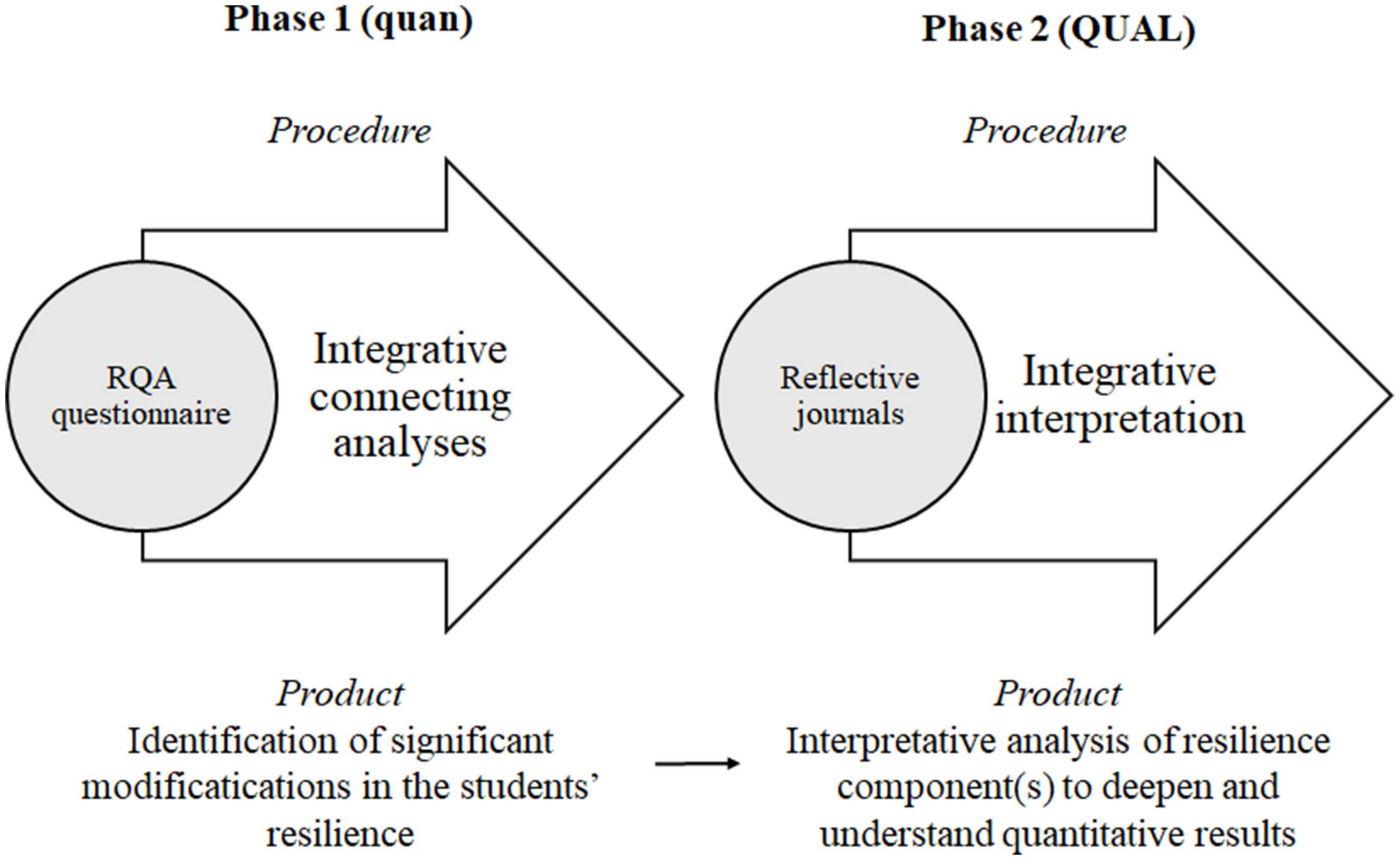
Explanatory sequential mixed-methods research design.

The research used a mixed methodological approach based on an explanatory sequential design, as it aimed to understand a process as complex as the development of resilience, in which a multitude of factors intervene. According to Plano-Clark (2019), the combination of quantitative and qualitative methods in educational research allows for a more complete and holistic understanding of educational phenomena, while benefiting from the strengths of both approaches. The study follows an explanatory sequential mixed methods design divided into two phases, giving more relevance to the qualitative part of the study (Figure 1). The first phase adopts a quasi-experimental quantitative approach within the positivist paradigm. A pre-post data analysis was carried out comparing the results obtained in the Resilience Questionnaire for Adults (RQA) by Alonso-Tapia et al. (2017). Informed by these results, the second phase opts for a qualitative approach from the interpretive paradigm. In this phase, personal reflection journals completed by the participating students are used in order to delve deeper into their experiences and understand how the dimensions of the resilience construct might have changed. All in all, this means that the study entailed an initial quantitative part to check for differences and, in case modifications were found, a subsequent qualitative component, with greater research prominence, would come into action.

Meaningful integration of quantitative and qualitative data is critical within mixed methods studies (Plano-Clark, 2019). In order to achieve such integration within this research, the four strategies to facilitate meaningful integration established by Plano-Clark (2019) were adopted:

1. Asking integrative mixed methods research questions. In this sense, in case the hypothesis posed is confirmed, the research question posed in the second phase (qualitative part of the study) aims to deepen and understand the results obtained in the first phase (quantitative part).
2. Aligning the quantitative and qualitative data sources. Both information sources are focused on the resilience construct.
3. Identifying points of integration. The visual display (Figure 1) illustrates the moments in the study when the two methods (quantitative and qualitative) come into contact with each other.
4. Developing joint displays and mixed interpretations. The results section presents a joint display in the form of a table to explicitly relate quantitative and qualitative information.

This work is part of a research project (UJI-A2022-11) and has the approval of the ethics committee of Universitat Jaume I (CEISH/47/2022). In addition, the ethical guidelines proposed in the Declaration of Helsinki have been respected (Word Medical Association, 2013).

### Participants of the study

54 students enrolled in the Basic Physical Education subject of the second year of the degree in Physical Activity and Sports Sciences at the Universitat Jaume I (Castellón de la Plana, Spain) participated in this study. They were selected using a non-probabilistic convenience sampling technique. Ultimately, a total of 31 students answered both the pre- and post-test accurately (29% women and 71% men).

### Service-Learning program

#### Contextualization

The SL program was carried out in the 22/23 academic year in the subject “Basic Physical Education” (ES2019). The objective of this subject lies in the study and practical application of the relationships established between human motor skills and perceptive faculties. In the SL program, the students designed and applied motor sessions aimed at children with functional diversity with different degrees of affectation and in a situation of social exclusion or vulnerability. Table 2 presents a summary table with the essential information of the program.

**Table 2:** Summary table of the Service-Learning program.

Service-Learning program				
Reference subject	Specific content worked on in the interventions	Academic objectives	Service developed	Service objectives
Basic Physical Education. 2nd year of the degree “Physical Activity and Sports Sciences,” taught in the second semester and compulsory (6 ECTS).	Motor and perceptual-motor skills, perception and representation of oneself and the outside environment, motor play, socialization through motor movement.	To develop the ability to work on the motor skills of students with educational needs through physical-sports sessions. To promote the inclusion of students with functional diversity.	To design and implement motor skills sessions.	To improve the motor and socialization areas of children at risk. To improve the educational inclusion of children with functional diversity.

Own elaboration.

In Castellón de la Plana, the offer of free and accessible physical-sports activities for children with diverse individual characteristics and socioeconomic situations is almost non-existent, with private options being an unaffordable economic cost for many families. Therefore, the common challenge for each group was to respond to this lack by creating a non-clinical space that would address the needs of these children appropriately.

#### Participant social partners

In addition to the university students, four social entities participated in the program. Each group of students partnered with one of these entities, which they selected bearing in mind their motivation, interests and schedule possibilities. Below, the various partner entities that took part in the program and the characteristics of the children they attend, who participated in the service, are specified.

- Association of Parents of People Affected by Attention Deficit and Hyperactivity (APADAHCAS) of the province of Castellón. Its purposes are guiding, researching and collaborating with centers or professionals dedicated to the study of attention deficit and hyperactivity.
- Cruz Roja (non-governmental organization). Specifically, the program collaborated with the “School Success Project,” which aims to make education accessible to children in situations of social exclusion or vulnerability.
- Castellvell Special Education Center. This special education school has students with functional diversity where the students are diverse in terms of age, type of affectation and its severity.
- Penyeta Roja Special Education Center. This special education school has students with functional diversity where the students are diverse in terms of age, type of affectation and its severity.

#### Organization and teaching issues

The design and implementation of the SL program followed the model of Chiva-Bartoll and Fernández-Rio (2022), which specifies four phases: preparation (preliminary meetings to prepare the program), planning (initial contact between university students and collaborating entities), execution (development of the sessions) and evaluation and recognition (data collection and events with the agents involved in which relationships and learning are strengthened). The university students, organized into 9 groups of 3–7 members, planned and led practical sessions that combined direct interaction with groups of children and the application of knowledge of the subject, ensuring the technical usefulness of the project. To monitor the program, each group completed a reflective journal in which there was an individual part to reflect on their personal experience. In addition, effective feedback strategies were used in the review of these journals and in post-session reflections. In these post-session reflections, the teachers and staff of the partner entities discussed with the students upon their teaching practice. The aim was to analyze students’ development and guide them to identify issues that had arisen and ways to improve their future practice. Members of the partner entities took part in these reflections in order to enrich them, given that they offered an alternative perspective and shared their experience working with the group of children.

### Data gathering instruments

In terms of the quantitative approach, the instrument administered to measure the dimensions of resilience was the RQA (Alonso-Tapia et al., 2017), since this questionnaire has been validated for the general adult population. The reliability of the RQA dimension scores was estimated using McDonald’s *ω* coefficient. The reliability tests were excellent and those of the indicators were acceptable to excellent. Only sensitivity had *ω* < 0.70. Most importantly, the prediction values (*γ*) were all significant. This is a 36-item Likert scale, which is rated from 1 to 5, with 1 indicating “totally disagree” and 5 representing “totally agree.” This questionnaire is structured into different dimensions, which are related to each of the resilience components (Prince-Embury, 2006; Prince-Embury and Saklofske, 2014). Regarding the component of “Mastery,” the first dimension is Optimism and measures the tendency to expect favorable future results; the second is Self-efficacy and measures optimistic beliefs about one’s own ability to face obstacles; the third is Adaptability and measures the ability to be receptive to criticism, learn from mistakes and ask for help. Moving now to the component of Relational sense the dimension of Trust measures the degree to which others are perceived as reliable in order to show authenticity in these relationships; the next dimension is Support, linked to the individual’s belief that there are other people they can turn to when faced with adversity. Another dimension is Comfort and measures the degree to which a person can be in the presence of others without feeling uncomfortable; while the dimension of Tolerance measures the person’s belief that they can safely express their differences. Finally, the components connected to Emotional reactivity are Sensitivity, that measures the emotional reactivity of individuals to environmental demands. Recovery, that measures the ability to recover from emotional arousal or disturbance of balance. And Deterioration which measures the degree to which a person is able to maintain emotional balance when excited.

As for the qualitative approach, the information provided by the university students in their reflection journals was analyzed. The reflection journals are written narratives that provide information on how social actors produce, represent and contextualize their personal experience and knowledge (Coffey and Atkinson, 2005). It is a consolidated tool used in previous SL studies in higher education developed in the field of PE (Maravé-Vivas et al., 2023b; Ruiz-Montero et al., 2019) given that reflective journals are a rich learning source for the Service-Learning classroom (Deeley, 2022). These reflection journals had a group section and an individual part. In the group part students had to describe and reflect upon the motor games sessions that they initially designed, as well as the modifications that they introduced during the implementation stage. It also compiled information regarding the difficulties that arose during the intervention and how the students faced them. Regarding the individual section of the journal, students were to share their personal experiences during the different phases of the project (before, during and after).

## Results

This research, that aimed to analyse the effects of a SL program on the resilience of the participant students, used a mixed approach in order to take profit of the complementary nature of quantitative and qualitative approaches. First, we present the quantitative part of the study. Particularly, the data analyses carried out are specified and the quantitative results on the changes in the resilience variables are presented. Next, the qualitative part of the study is presented. It shares an in-depth examination of the resilience variables to gain insight of the quantitative results previously obtained.

### Phase 1: data analysis and quantitative results

The hypothesis guiding this part of this study was: the application of SL in a university subject of the Physical Activity and Sports Sciences degree will produce significant improvements (*p* < 0.05) in the resilience levels of the participating students. In this phase, data were processed with the IBM SPSS Statistics software (version 25) using descriptive and inferential statistics. The normality of the sample was checked by homoscedasticity and distribution analysis (Kolmogorov–Smirnov). Once normality was checked, parametric tests (Student’s t) were performed for related samples. Parametric tests were chosen for the comparison of the pre- and post-test results given that the distribution of the data was normal and met the assumption of homoscedasticity. Specifically, the Student *t*-test was used to compare means for related samples (Table 3).

**Table 3:** Paired samples test.

	Paired differences					*t*	gl	Significance	
	Average	Standard deviation	Mean standard error	95% confidence interval of the difference				*p* one-factor	*p* two-factor
				Lowest	Highest				
Global average pretest Global average post-test	−0.07796	0.18676	0.03354	−0.14646	−0.00945	−2.324	30	0.014	0.027
Average adaptability pretest Average adaptability post-test	−0.21774	0.61827	0.11104	−0.44453	0.00904	−1.961	30	0.030	0.059
Average tolerance pretest Average tolerance post-test	−0.16935	0.51392	0.09230	−0.35786	0.01915	−1.835	30	0.038	0.076

Own elaboration.

Overall, considering the RQA as a whole, a significant difference was obtained in the post-test, *t*(31) = −07796, *p* < 0.05, with a medium effect size. There were differences in the mean scores of the post-test in the two dimensions of the RQA, reaching significance in the adaptability and tolerance subcomponents too. Given that the hypothesis posed for the quantitative part of the study was confirmed (there were significant modifications in the resilience levels), we then moved to the second part of the research in order to dive deeper into understanding the nuances and mechanisms that explain these quantitative results.

### Phase 2: data analysis and qualitative results

The research question guiding this part of this study was: How does SL affect the dimensions of resilience? Bearing in mind the results of the quantitative phase of the study (evincing a significant difference in the RQA as a whole), the analysis of the qualitative part focused on the three components of resilience, given that the whole construct had been significantly modified in the first phase.

The content analysis of the qualitative data followed the phases proposed by Patton (2002): preparing the data, defining the unit of analysis, developing categories and creating a coding scheme, testing the coding scheme on a sample of text, coding the entire text and evaluating the coherence of the coding. The research team applied the deductive method, identified units of meaning and coded them according to the theoretical categories defined by Prince-Embury (2006) and Prince-Embury and Saklofske (2014): mastery, relational sense and emotional reactivity.

The article follows the rigor criteria of qualitative research established by Noreña et al. (2012), ensuring that the findings faithfully reflect the perceptions of the students. It also describes in detail the program, the participants and the reflection processes carried out; with anonymized and coded data indicating the number of the student to whom the individual journal belongs to, the group to which they belong to and the acronym of the partner entity. In addition, the abbreviation assigned to the dimension of resilience to which it refers to appears in brackets, as well as a number that indicates the order in which the quote is found within the reflection journal. An example would be the following: A.1. C.I (G7. P.R) [OP. 1]. The quotes presented below are representative and are identified by their origin. In addition, information triangulation was used when collecting data in various contexts and with different participants (Denzin, 2012).

Table 4 shows the two types of data gathered. It presents how the quantitative part of the study enabled the development of the qualitative phase. In addition, regarding the qualitative results, the table summarizes the coding scheme, linking resilience components and dimensions to specific student reflections.

**Table 4:** Overview of the results.

	QUAN part	QUAL part		
	RQA questionnaire	Categories (resilience components)	Codes (resilience dimensions)	Example of a quote
Resilience	Significant differences	Mastery	Optimism	A.48. C.I (G7. P.R) [OP. 1]
			Self-efficacy	A.2. C.I (G4. P.R) [AUT. 1]
			Adaptability	A.4. C.I (G9. APA) [ADAP. 2]
		Relational sense	Sense of trust	A.48. C.I (G7. P.R) [CONF. 1]
			Perception of access to support	A.15. C.I (G2. C.V) [AP. 1]
			Comfort	A.7. C.I (G4. P.R) [COM. 1]
			Tolerance	A.9. C.I (G3. C.V) [TOL. 1]
		Emotional reactivity	Recovery	A.48. C.I (G7. P.R) [REC. 1]
			Sensitivity	A.13. C.I (G7. P.R) [SEN. 5]
			Deterioration	A.13. C.I (G7. P.R) [DET. 1]

Own elaboration.

The qualitative results obtained from the analysis of the reflection journals completed by the students are presented below. The analysis of these reflection journals focuses on identifying explanations and reasoning that respond to how resilience has changed, given that a statistically significant overall improvement was detected. Resilience consists of three global components as reflected in Table 1. Their definition is recalled, and the results are presented addressing each of these.

Regarding the sense of mastery, it refers to the innate capacity of people to solve problems and face adversities in life, and includes the dimensions of optimism, self-efficacy and adaptability. In this sense, one student states:

**A.48. C.I (G7. P.R) [OP. 1]** “If I were to teach a group of children now, I would know very well how to lead the class and plan activities, partly thanks to the confidence acquired in the SL sessions. If I had not participated in this program, the day I met a child with special needs I would not know how to act, but after having been with the children of Penyeta I think I would know how to deal with them.”

The quote reflects how, after the intervention, there has been an improvement in the ability to organize sessions for students in general and with functional diversity in particular, improving their self-efficacy and adaptability. The experience in a different environment, together with learning from mistakes, strengthens skills, encourages coexistence and reduces prejudices, generating optimism to work with this group in the future.

One of the current and historical objectives of learning is that it is transferable to real-life situations. In this case, a student points out:

**A.2. C.I (G4. P.R) [AUT. 1]** “With the SL I have had to adapt to them and know how to get out of situations or momentary changes and for this I have had to use what I have learned.”

The quote offers clues to the conscious level that what has been learned has been useful in dealing with certain adversities. The SL program has facilitated the development of competency-based learning, as it has allowed students to apply what they have learned and develop the ability to adapt to changing and stressful situations. This influences their degree of optimism, self-efficacy and adaptability, currently perceiving themselves as people capable of dealing with similar situations.

Regarding the variation observed in the relational sense of the students, this refers to the way in which the individual relates to others in adverse situations, and incorporates the dimensions of sense of trust, access to support and comfort. In this context, a student states:

**A.48. C.I (G7. P.R) [CONF. 1]** “My feeling towards them has changed: because I have realized that they can also be happy despite their pathology; and because I have become friends with my students, something that a few months ago I would not have said, because I did not know of any cases like these.”

This quote shows that, despite prejudices and social invisibility toward diversity, the intervention allowed them to overcome preconceived ideas, manage discomfort and establish friendships that generated an increase in trust and acceptance. In addition, university students strengthened social ties with both the group and educational professionals, who acted as a support network, helping them to reflect and manage situations related to diversity. The latter is reflected in the following quote:

**A.15. C.I (G2. C.V) [AP. 1]** “They welcomed us very well and provided us with any type of help, in addition, at all times they were aware that we had never worked in this way and they guided us.”

Regarding emotional reactivity, defined as the individual’s capacity to modulate and regulate their emotional responses, which covers the dimensions of sensitivity, recovery and deterioration, it is worth highlighting the following quote:

**A.13. C.I (G7. P.R) [SEN. 5]** “My grandmother suffers from Alzheimer’s. After spending the afternoon with her, sadness and negative emotions take hold of me. And the fact is that on many occasions, the behavior and life that my grandmother experiences today are very similar to those of some of our students. And this leaves a bad taste in your mouth, since my grandmother has lived a long time, and these conditions are only for the final stage of life, but for many people with special characteristics they are conditioned from their initial stages; and the worst thing is that, generally, these conditions will not change throughout their life.”

After reflecting, this student compares their current personal situation with that of the students in the group, this being a common practice among people who undergo these experiences. The condition of the group has an impact on the sensitivity of the student, helping to identify and value positive aspects of their own life and the lives of others. It also develops the ability to reorder priorities and manage day-to-day problems, giving them the importance they deserve.

A very present topic in the reflection journals is happiness. Everyone wants to be happy, and to do so they set goals to achieve it (financial solvency, physical and mental health, social relationships, etc.). Before the intervention, a large part of the students believed that students with educational needs could not be happy. After the sessions, the students have been able to confirm that this is not true, the only thing that changes are the goals to achieve, which are adjusted to the individual capacities of each person. In this sense, one student stated that:

**A.48. C.I (G7. P.R) [REC. 1]** “[…] Another prejudice was that these children lived in sadness and could not be happy, but seeing how they enjoy the sessions with us made me realize that I was wrong. I also thought that disability was an illness, but that is not the case, disability is a condition and that does not mean that they have to be cured, but that they should live as they are.”

This quote is an example of how the participating group enjoyed the sessions and how the university students contributed to their happiness. This aspect is linked to the dimension of recovery, because at the moment in which the university students know and accept the situation of the group, they are able to reflect on how their actions can influence it and help it. Thus, although the condition of the people in the group is permanent, their evolution is not, they can all improve within their possibilities.

## Discussion

It is worth mentioning that this work introduces a new perspective in terms of research into the effects of SL on the resilience levels of university students. Consequently, the existing literature found in reference to the promotion of resilience, as a general concept, in the educational field has been quite limited. For this reason, the discussion focuses on the effects of SL linked to the indicators of the three main components that constitute the global construct of resilience. The research hypothesis questions whether the application of SL in a university subject of the Physical Activity and Sports Sciences degree will produce significant improvements (*p* < 0.05) in the resilience levels of the participating students. In line with the analysis of quantitative data carried out, there are significant differences favoring the RQA post-test that indicate that the hypothesis has been fulfilled. These results are in line with what Mayor Paredes (2018) and Monge-Hernández et al. (2020) stated, who points out that SL experiences favor the comprehensive development of students, allowing the acquisition of knowledge, skills, attitudes and essential values to face challenges in different areas. This result is also consistent with the work of Fernández-Cabrera et al. (2021), which highlights that SL, by delegating responsibilities to participants, promotes their empowerment and resilience. The connection between academic learning and community service offered by SL programs strengthens adaptation and overcoming adversities (Bringle et al., 2006; Shephard and Egan, 2018), central elements in the definition of resilience. Thus, this article confirms that the SL pedagogical model could be an effective tool to promote resilience in university students.

The results of the quantitative and qualitative analysis were complemented to meet the general objective of the research. The use of a mixed research approach offers a greater degree of understanding of the phenomenon studied (Chiva-Bartoll et al., 2019). In this case, having already confirmed a quantitative modification at a global level of the RQA, it is convenient to interpret the areas of improvement from a qualitative research approach, to explore what has improved and how it has been achieved. Thus, answering the question posed at the beginning: How will SL affect the dimensions of resilience? Regarding the sense of mastery, the qualitative results point to an improvement linked to the perception that students have about their skills, as well as their ability to learn from mistakes and adapt to an unknown and changing context. These areas of improvement directly influence the resilience of students, given that being resilient implies not only resistance to difficult situations, but also the process of learning and growing from those challenging experiences (Fletcher and Sarkar, 2013). That is, the ability to mobilize internal and external resources that allow one to face and overcome adversities (Bonanno et al., 2004). Studies such as those by Morillo-Flores et al. (2022) and Correa Valencia et al. (2024) explain how the interaction between theory and practice when applying SL promotes autonomous learning, creativity and adaptation to diverse situations. This means that there is an improvement in the students’ self-perception and self-confidence as well as their sensitivity to other realities (Martínez-Sanz and Stolle, 2020). In this sense, authors such as Yorio and Ye (2012) and Cañadas (2021) state that people who get involved in services for and with others establish social interactions that contribute to their own self-definition and self-perception, generating improvements in their self-efficacy and security. Based on this finding, adaptability and self-efficacy are indicators that are part of the resilience construct (Bryan et al., 2015; Keye and Pidgeon, 2013; Masten and Motti-Stefanidi, 2020), as they allow people to learn from a challenging context and develop attitudes and strategies to adapt their actions to its demands.

In reference to the changes perceived in the relational sense, it is observed how the students have expanded their support network through the creation of friendships with the group and with the professionals involved in it. Likewise, the SL program has allowed them to overcome their initial discomfort and get to know students with functional diversity, thus overcoming certain prejudices. This is in line with what Deeley (2016) postulated, who indicates that experiences that produce discomfort in participants can be a source of learning. In reference to this finding, authors such as Abellán et al. (2021) and Tindall et al. (2016), showed that participation in SL projects implies a significant improvement in the level of confidence of the members when it comes to attending to and including the group, thus improving their attitudes toward it, particularly empathy. In relation to this, Rodríguez-Izquierdo (2019) indicates that SL not only focuses on meaningful learning but also seeks social transformation through community service. This enrichment of the curriculum makes SL to be an adequate approach for inclusive practices (Mella-Núñez et al., 2021; Tripon, 2024). This is consistent with several studies that observed the transformation of initial assumptions and stereotypes as a result of SL experiences (Domangue and Carson, 2008; Maravé-Vivas et al., 2023a; Pérez-Ordás et al., 2021). The mental restructuring of preconceived notions involves leaving one’s comfort zone and overcoming the initial discomfort derived from ignorance and misinformation. According to Tripon (2024), SL encourages students to examine their assumptions and biases about the partner group, learning to respect their differences and thus acquiring cultural insights that will enable them to create more inclusive learning environments. According to Prince-Embury (2008), this ability to overcome change, get to know the group and relate in adverse and unpredictable situations, even establishing bonds of friendship, is part of the resilience construct. This is consistent with what Ungar and Theron (2020) say, as they state that resilience levels increase when there is knowledge and sensitivity toward the contextual and cultural dynamics of the group.

Regarding the emotional reactivity factor, the main changes in students are linked to the emotional impact that the intervention has on them. In this sense, students tend to compare the situation of the group with their own, experiencing different sensations. It has been observed how this sensitivity has allowed students to analyze the situation, recover from the emotional shock and join forces to help the group. In line with Prince-Embury (2008), the ability to regulate their emotional reactions, become sensitive to others and recover from the emotional shock caused by the differences between the two, are part of the resilience construct. In this sense, García-Rico et al. (2020) and Morillo-Flores et al. (2022) argue that SL contributes to professional training based on social justice and the ability to commit to the transformation of situations of inequality, segregation or invisibility that different sectors of the community face. Likewise, authors such as Chiva-Bartoll et al. (2020) and Simsek (2020) point out that SL experiences influence the construction of the identity and personality of the participants, facilitating the acquisition of key competencies in their professional future, such as leadership and problem-solving skills (Tudela et al., 2021), and the ability to reflect on their role as agents of change (Cano and García-Martín, 2021). This is consistent with the findings of Min et al. (2024), who state that all learning linked to knowledge of one’s own identity and control of emotions enables the individual to improve their self-regulation, empathy and social connection, factors closely linked to resilience (Masten, 2001; Prince-Embury and Saklofske, 2014).

In light of the findings obtained, it can be concluded that the SL experience allowed university students to interact with socially invisible groups, transforming their perceptions and understanding of them. This pedagogical approach facilitated the connection between theory and practice, promoting competency-based learning that includes useful knowledge, skills, and attitudes for facing real-life challenges. Furthermore, according to the quantitative and qualitative results, SL favored the development of resilience, especially in adaptation and tolerance to differences; consolidating itself as a pedagogical model that could be effective in promoting resilience in university students.

In this sense, it is important to note that the SL program carried out could be implemented in other higher education degrees, supporting previous literature sharing experiences and research that promote its use in different educational disciplines (Salam et al., 2019; Sotelino-Losada et al., 2021). The learning developed by the students who engage in SL is relevant for undergraduates in general, so it would be interesting to replicate similar programs in other contexts and degrees. To do so, it is necessary to exhaustively describe the program (academic and service objectives, role model, type of service, involvement of collaborating entities, functions of all committed agents, student reflection instruments, timing…), as we did in the present study. That is, all the elements that build a program must be clearly defined to ensure that certain quality criteria are met when planning and designing SL programs that aim to benefit all agents involved. In addition, to promote the use of SL in other disciplines, it would be interesting to move toward the institutionalization of SL at the university level. In this way, the university can provide all stakeholders with a reference SL model specific to their context and include pedagogical and research training as well as personal support resources, among other issues. Given the multiple academic, personal and social benefits that SL can generate, the university leaders, as well as those in charge of the design and modification of the different study programs, could more easily include SL within different initiatives at the university level as a transversal, regardless of the discipline of study.

The results presented here should be taken with caution, since this study is not without limitations. For instance, the number of participants prevents us from generalizing the results obtained. However, the mixed methods approach used enabled us to not only rely on quantitative data, but qualitative information was used to explain it and enhance our understanding of the results obtained in the first phase of the study. In addition, the SL program had a limited duration, but semester-long interventions are widespread in SL research in the PE (Cañadas, 2021; Chiva-Bartoll et al., 2019; Ruiz-Montero et al., 2019). Finally, we acknowledge that that there is a potential social desirability in gathering qualitative information through reflective journals. Nevertheless, these instruments are still widely used in this field of research (i.e., Carrington, 2011; Carrington and Selva, 2010; Deeley, 2022; Eutsler et al., 2023). Derived from these ideas, several lines of future work are identified: (1) carrying out studies with lager sample sizes in order to be able to generalize the results, (2) implementing longer SL programs in order to compare whether the length of the intervention has a significant impact on resilience components, and (3) to gather additional qualitative information (i.e., interviews) and including the perceptions of other agents involved in the SL program (i.e., university teachers, community partners).

## Data Availability

The datasets presented in this article are not readily available because they are part of a much larger research project involving several doctoral theses in progress. Requests to access the datasets should be directed to marave@uji.es.

## References

[ref1] Abellán J. Segovia Y. Gutiérrez D. López L. M. G. (2021). Sensibilización hacia la discapacidad a través de un programa integrado de Educación Deportiva y Aprendizaje-Servicio. Retos 43, 477–487. doi: 10.47197/retos.v43i0.86625

[ref2] Alonso-Tapia J. Garrido-Hernansaiz H. Rodríguez-Rey R. Ruiz M. Á. Nieto C. (2017). Personal factors underlying resilience: development and validation of the resiliency questionnaire for adults. Int. J. Ment. Health Promot. 19, 104–117. doi: 10.1080/14623730.2017.1297248

[ref3] Bauman Z. (2003). Modernidad líquida. Buenos Aires: Fondo de Cultura Económica.

[ref4] Bonanno G. A. Wortman C. B. Nesse R. M. (2004). Prospective patterns of resilience and maladjustment during widowhood. Psychol. Aging 19, 260–271. doi: 10.1037/0882-7974.19.2.260, PMID: 15222819

[ref5] Brewer M. L. Van Kessel G. Sanderson B. Naumann F. Lane M. Reubenson A.. (2019). Resilience in higher education students: a scoping review. High. Educ. Res. Dev. 38, 1105–1120. doi: 10.1080/07294360.2019.1626810

[ref6] Bringle R. G. Clayton P. H. (2021). Civic learning: A sine qua non of service-learning. Front. Educ. 6:39. doi: 10.3389/feduc.2021.606443

[ref7] Bringle R. G. Hatcher J. A. McIntosh R. E. (2006). Analyzing Morton's typology of service paradigms and integrity. Michigan J. Commun. Serv. Learn. 13, 5–15. http://hdl.handle.net/2027/spo.3239521.0013.101

[ref8] Bryan C. J. Ray-Sannerud B. Heron E. A. (2015). Psychological flexibility as a dimension of resilience for posttraumatic stress, depression, and risk for suicidal ideation among air force personnel. J. Contextual Behav. Sci. 4, 263–268. doi: 10.1016/j.jcbs.2015.10.002

[ref9] Cañadas L. (2021). Aprendizaje-Servicio universitario en contextos de actividad física, educación física y deporte: una revisión sistemática. Educ. Pesqui. 47:7446. doi: 10.1590/s1678-4634202147237446, PMID: 40053013

[ref10] Cano E. B. García-Martín J. (2021). El impacto del aprendizaje-servicio (ApS) en diversas variables psicoeducativas del alumnado universitario: las actitudes cívicas, el pensamiento crítico, las habilidades de trabajo en grupo, la empatía y el autoconcepto. Una revisión sistemática. Rev. Complut. Educ. 32, 639–649. doi: 10.5209/rced.70939

[ref11] Carrington S. (2011). Service-learning within higher education: Rhizomatic interconnections between university and the real world. Aust. J. Teach. Educ. 36, 1–14. doi: 10.14221/ajte.2011v36n6.3

[ref12] Carrington S. Selva G. (2010). Critical social theory and transformative learning: evidence in pre-service teachers’ service-learning reflection logs. High. Educ. Res. Dev. 29, 45–57. doi: 10.1080/07294360903421384

[ref9001] Chambers D. Lavery S. (2022). International service learning: benefits, challenges and experiences of pre-service teachers. Asia-Pac. J. Teach. Educ. 50, 498–514. doi: 10.1080/1359866X.2022.2050355

[ref13] Chiva-Bartoll O. Fernández-Rio J. (2022). Advocating for service-learning as a pedagogical model in physical education: towards an activist and transformative approach. Phys. Educ. Sport Pedagog. 27, 545–558. doi: 10.1080/17408989.2021.1911981

[ref14] Chiva-Bartoll O. Gil-Gómez J. (2018). Aprendizaje-servicio universitario: modelos de intervención e investigación en la formación inicial docente. Barcelona: Ediciones Octaedro.

[ref15] Chiva-Bartoll O. Montero P. J. R. Capella-Peris C. Salvador-García C. (2020). Effects of service learning on physical education teacher education students’ subjective happiness, prosocial behavior, and professional learning. Front. Psychol. 11:331. doi: 10.3389/fpsyg.2020.00331, PMID: 32226402 PMC7080846

[ref16] Chiva-Bartoll O. Ruiz-Montero P. Martín Moya R. Pérez López I. Giles Girela J. García-Suárez J.. (2019). Aprendizaje-servicio universitario en educación física y ciencias del deporte: una revisión sistemática. Rev. Complut. Educ. 30, 1147–1164. doi: 10.5209/rced.60191

[ref17] Coffey A. Atkinson P. (2005). Encontrar el sentido a los datos cualitativos: estrategias complementarias de investigación. Medellín: Universidad de Antioquia.

[ref18] Correa Valencia M. Gordillo Suarez M. Sepúlveda-Salcedo L. S. (2024). Aprendizaje-Servicio en la formación de ingenieros: caso de estudio. Eur. Public Soc. Innov. Rev. 10, 1–14. doi: 10.31637/epsir-2025-547

[ref19] Deeley S. J. (2016). El Aprendizaje-Servicio en educación superior. Teoría, práctica y perspectiva crítica. Madrid, Spain: Narcea Ediciones.

[ref20] Deeley S. (2022). Assessment and service-learning in higher education. London: Palgrave Macmillan.

[ref21] Denzin N. K. (2012). Triangulation 2.0. J. Mixed Methods Res. 6, 80–88. doi: 10.1177/1558689812437186

[ref22] Domangue E. Carson R. L. (2008). Preparing culturally competent teachers: service-learning and physical education teacher education. J. Teach. Phys. Educ. 27, 347–367. doi: 10.1123/jtpe.27.3.347

[ref23] Eutsler L. Naik M. Peecksen S. Branton R. (2023). Impact of inquiry portfolios within a service-learning literacy field experience on preservice teachers’ knowledge growth and GPA. Teach. Teach. Educ. 124:104032. doi: 10.1016/j.tate.2023.104032

[ref24] Fazel M. Patel V. Thomas S. Tol W. (2014). Mental health interventions in schools in low-income and middle-income countries. Lancet Psychiatry 1, 388–398. doi: 10.1016/S2215-0366(14)70357-8, PMID: 26361001

[ref25] Fernández-Cabrera J. M. Gómez-Rijo A. Jiménez-Jiménez F. Ríos-Hernández M. (2021). El encuentro socio-deportivo como experiencia de aprendizaje-servicio universitario en dos centros de internamiento educativo de menores. Contextos Educ. 27, 117–134. doi: 10.18172/con.4653

[ref26] Fletcher D. Sarkar M. (2013). Psychological resilience. Eur. Psychol. 18, 12–23. doi: 10.1027/1016-9040/a000124

[ref27] García-Rico L. Carter-Thuillier B. Santos-Pastor M. L. Martínez-Muñoz L. F. (2020). Formar profesores de educación física para la justicia social: efectos del aprendizaje-servicio en estudiantes chilenos y españoles. Rev. Int. Educ. para Justicia Soc. 9, 29–47. doi: 10.15366/riejs2020.9.2.002

[ref28] Gil-Gómez J. Martí-Contreras J. (2024). Las prácticas curriculares en alternancia: propuestas para el ámbito universitario. Barcelona: Editorial Graó.

[ref29] Gillham J. E. Reivich K. Freres D. R. Chaplin T. M. Shatté A. Samuels B.. (2007). School-based prevention of depressive symptoms: A randomized controlled study of the effectiveness and specificity of the PeNn resiliency program. J. Consult. Clin. Psychol. 75, 9–19. doi: 10.1037/0022-006X.75.1.9, PMID: 17295559 PMC4469032

[ref30] Keye M. D. Pidgeon A. M. (2013). Investigation of the relationship between resilience, mindfulness, and academic self-efficacy. Open J. Soc. Sci. 1, 1–4. doi: 10.4236/jss.2013.16001

[ref32] Maravé-Vivas M. Gil-Gómez J. Moliner García O. Capella-Peris C. (2023a). Service-learning and physical education in preservice teacher training: toward the development of civic skills and attitudes. J. Teach. Phys. Educ. 42, 631–639. doi: 10.1123/jtpe.2022-0094

[ref33] Maravé-Vivas M. Salvador-García C. Capella-Peris C. Gil-Gómez J. (2023b). Service-learning and motor skills in initial teacher training: doubling down on inclusive education. Apunts. Educ. Fís. Deporte 152, 82–89. doi: 10.5672/apunts.2014-0983.es.(2023/2).152.09

[ref34] Martínez-Sanz R. Stolle P. D. (2020). Comunicación corporativa y educación en valores en el aula universitaria. Un proyecto de Aprendizaje-servicio (APS). OBETS Rev. Cienc. Soc. 15, 563–588. doi: 10.14198/OBETS2020.15.2.07

[ref35] Masten A. S. (2001). Ordinary magic: resilience processes in development. Am. Psychol. 56, 227–238. doi: 10.1037/0003-066X.56.3.227, PMID: 11315249

[ref36] Masten A. S. (2014). Ordinary magic: resilience in development. Choice 52:52-2831. doi: 10.5860/CHOICE.187892, PMID: 17723383

[ref37] Masten A. S. Motti-Stefanidi F. (2020). Multisystem resilience for children and youth in disaster: reflections in the context of COVID−19. Advers. Resil. Sci. 1, 95–106. doi: 10.1007/s42844-020-00010-w, PMID: 32838305 PMC7314620

[ref38] Mayor Paredes D. (2018). Aprendizaje-Servicio: una práctica educativa innovadora que promueve el desarrollo de competencias del estudiantado universitario. Actualid. Invest. Educ. 18, 494–516. doi: 10.15517/aie.v18i3.34418

[ref39] Mella-Núñez Í. Quiroga-Carrillo A. Comesaña J. C. (2021). Aprendizaje-servicio y desarrollo cívico-social en titulaciones universitarias del ámbito educativo: preparando al alumnado para la práctica de una educación inclusiva. Educar 57, 363–377. doi: 10.5565/rev/educar.1241

[ref40] Min H. J. Park S. Lee S. Lee B. Kang M. Kwon M. J.. (2024). Building resilience and social–emotional competencies in elementary school students through a short-term intervention program based on the SEE learning curriculum. Behav. Sci. 14:458. doi: 10.3390/bs14060458, PMID: 38920790 PMC11200739

[ref41] Monge-Hernández C. Boni-Aristizábal A. Wilson-Strydom M. (2020). Analysis from the capabilities approach of the contributions of service learning for students of a south African university. Rev. Electron. Educ. 24, 43–66. doi: 10.15359/ree.24-3.3

[ref42] Morillo-Flores J. Menacho-Vargas I. Fuster-Guillén D. Tamashiro-Tamashiro J. (2022). Impact of service-learning in the training of university students. Int. J. Health Sci., 659–670. doi: 10.53730/ijhs.v6nS7.11220

[ref43] Noreña A. L. Alcaraz-Moreno N. Rojas J. G. Rebolledo-Malpica D. (2012). Aplicabilidad de los criterios de rigor y éticos en la investigación cualitativa. Aquichan 12, 263–274. doi: 10.5294/aqui.2012.12.3.5

[ref44] Patton M. Q. (2002). Qualitative research and evaluation methods. Thousand Oaks, CA: Sage.

[ref45] Pérez-Ordás R. Nuviala A. Grao-Cruces A. Fernandez-Martinez A. (2021). Implementing service-learning programs in physical education; teacher education as teaching and learning models for all the agents involved: a systematic review. Int. J. Environ. Res. Public Health 18:669. doi: 10.3390/ijerph18020669, PMID: 33466871 PMC7830582

[ref46] Plano-Clark V. L. (2019). Meaningful integration within mixed methods studies: identifying why, what, when, and how. Contemp. Educ. Psychol. 57, 106–111. doi: 10.1016/j.cedpsych.2019.01.007

[ref47] Prince-Embury S. (2006). Resiliency scales for children and adolescents. London: Pearson, 9–14.

[ref48] Prince-Embury S. (2008). The resiliency scales for children and adolescents, psychological symptoms, and clinical status in adolescents. Can. J. Sch. Psychol. 23, 41–56. doi: 10.1177/0829573508316592

[ref49] Prince-Embury S. Saklofske D. H. (2014). Resilience interventions for youth in diverse populations. Nueva York, NY: Springer.

[ref50] Reina R. Hemmelmayr I. Sierra-Marroquín B. (2016). Autoeficacia de profesores de educación física para la inclusión de alumnos con discapacidad y su relación con la formación y el contacto previo. Psychol. Soc. Educ. 8, 93–103. doi: 10.25115/psye.v8i2.455

[ref51] Resch K. Schrittesser I. (2023). Using the service-learning approach to bridge the gap between theory and practice in teacher education. Int. J. Incl. Educ. 27, 1118–1132. doi: 10.1080/13603116.2021.1882053

[ref52] Rodríguez-Izquierdo R. M. (2019). Aprendizaje Servicio y compromiso académico en Educación Superior. Rev. Psicodidáct. 25, 45–51. doi: 10.1016/j.psicod.2019.09.001, PMID: 40094570

[ref53] Ruiz-Montero P. J. Chiva-Bartoll O. Salvador-García C. Martín-Moya R. (2019). Service-learning with college students toward health-care of older adults: a systematic review. Int. J. Environ. Res. Public Health 16:4497. doi: 10.3390/ijerph16224497, PMID: 31739647 PMC6888558

[ref54] Sabouripour F. Roslan S. (2015). Resilience, optimism and social support among international students. Asian Soc. Sci. 11:159. doi: 10.5539/ass.v11n15p159

[ref55] Salam M. Awang Iskandar D. N. Ibrahim D. H. A. Farooq M. S. (2019). Service learning in higher education: A systematic literature review. Asia Pac. Educ. Rev. 20, 573–593. doi: 10.1007/s12564-019-09580-6

[ref56] Shephard K. Egan T. (2018). Higher education for professional and civic values: A critical review and analysis. Sustain. For. 10:4442. doi: 10.3390/su10124442

[ref57] Simsek M. H. (2020). The impact of service-learning on EFL teacher candidates’ academic and personal development. Eur. J. Educ. Res. 9, 1–17. doi: 10.12973/eu-jer.9.1.1

[ref58] Sohn E. (2022). Tackling the mental-health crisis in young people. Nature 608, S39–S41. doi: 10.1038/d41586-022-02206-9, PMID: 36002499

[ref59] Sotelino-Losada A. Arbues-Radigales E. Garcia-Docampo L. Gonzalez-Geraldo J. L. (2021). Service-Leaming in Europe. Dimensions and understanding from academic publication. Front. Educ. 6:604825. doi: 10.3389/feduc.2021.604825, PMID: 40093738

[ref60] Tindall D. Culhane M. Foley J. (2016). Pre-service teachers’ self-efficacy towards children with disabilities: an Irish perspective. Eur. J. Adapt. Phys. Act. 9, 27–39. doi: 10.5507/euj.2016.003

[ref61] Tripon C. (2024). Bridging horizons: exploring STEM students’ perspectives on service-learning and storytelling activities for community engagement and gender equality. Trends in High. Educ. 3, 324–341. doi: 10.3390/higheredu3020020

[ref62] Tudela J. M. O. Díaz-Pareja E. M. Cámara-Estrella Á. M. (2021). Futuros educadores, compromiso social y Aprendizaje-Servicio. Publica 51, 139–173. doi: 10.30827/publicaciones.v51i1.15746

[ref63] Ungar M. Theron L. (2020). Resilience and mental health: how multisystemic processes contribute to positive outcomes. Lancet Psychiatry 7, 441–448. doi: 10.1016/S2215-0366(19)30434-1, PMID: 31806473

[ref64] Word Medical Association. (2013). WMA Declaration of Helsinki -Ethical principles for medical research involving human subjects. Available online at: https://www.wma.net/what-we-do/medical-ethics/declaration-of-helsinki/ (Accessed January 10, 2022).

[ref65] Yorio P. L. Ye F. (2012). A meta-analysis on the effects of service-learning on the social, personal, and cognitive outcomes of learning. Acad. Manag. Learn. Edu. 11, 9–27. doi: 10.5465/amle.2010.0072

